# Supplementary figures and images for: Modeling dysbiosis of human NASH in mice: Loss of gut microbiome diversity and overgrowth of Erysipelotrichales
Source: PLoS One. 2021 Jan 4;16(1):e0244763. doi: 10.1371/journal.pone.0244763 (PMC7781477; doi:10.1371/journal.pone.0244763)

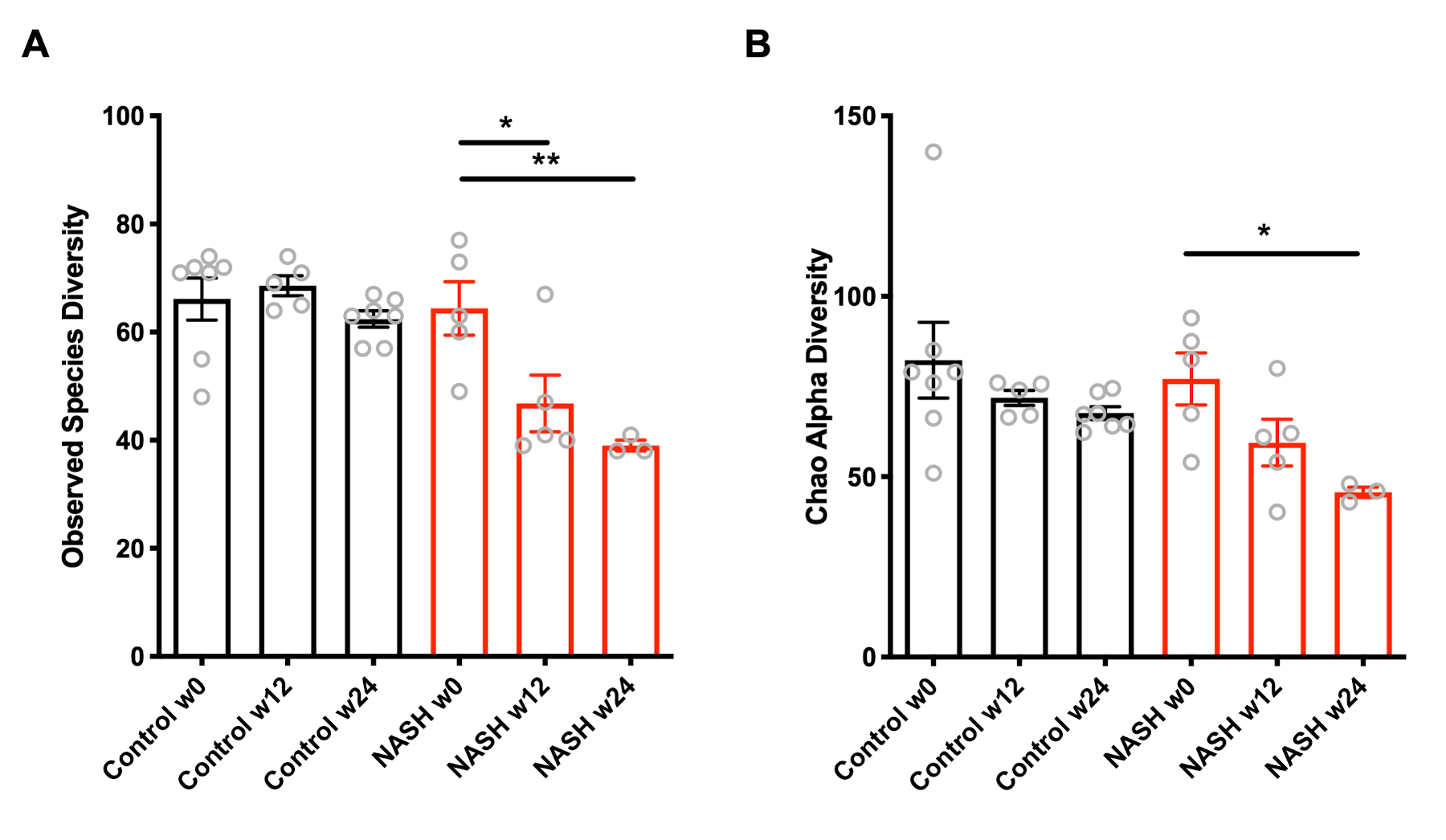

Supplement: S1 Fig — Alpha diversity calculated in control versus NASH mice using A) Observed Species or B) Chao Index. (TIFF) [file pone.0244763.s001.tiff]

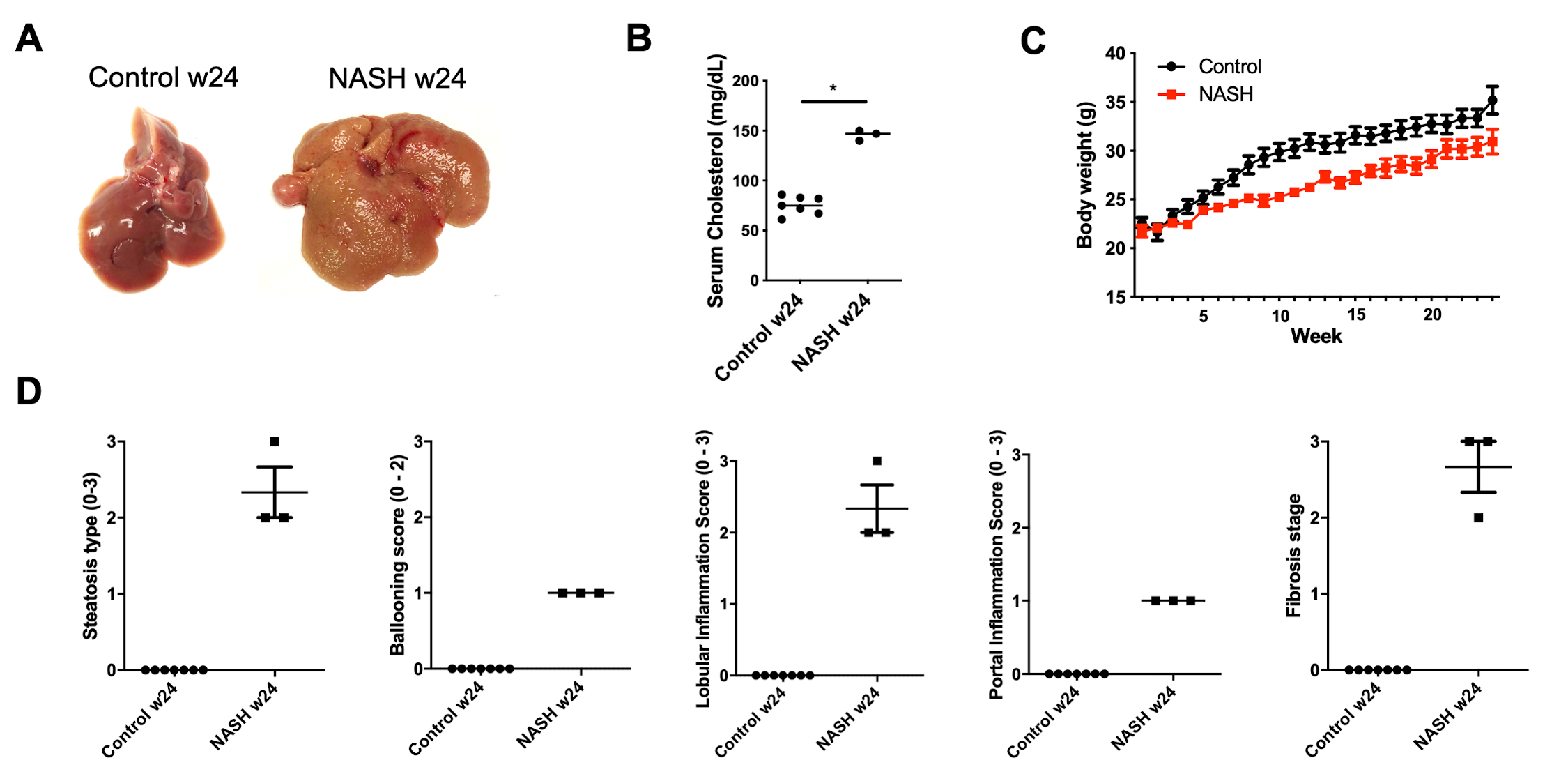

Supplement: S2 Fig — A) Images of whole liver collected from control or NASH mice at week 24. B) Serum cholesterol measurements at week 24. C) Mouse weights tracked weekly during the 24 week NASH protocol. D) Liver histology scored by a blinded pathologist. (TIFF) [file pone.0244763.s002.tiff]

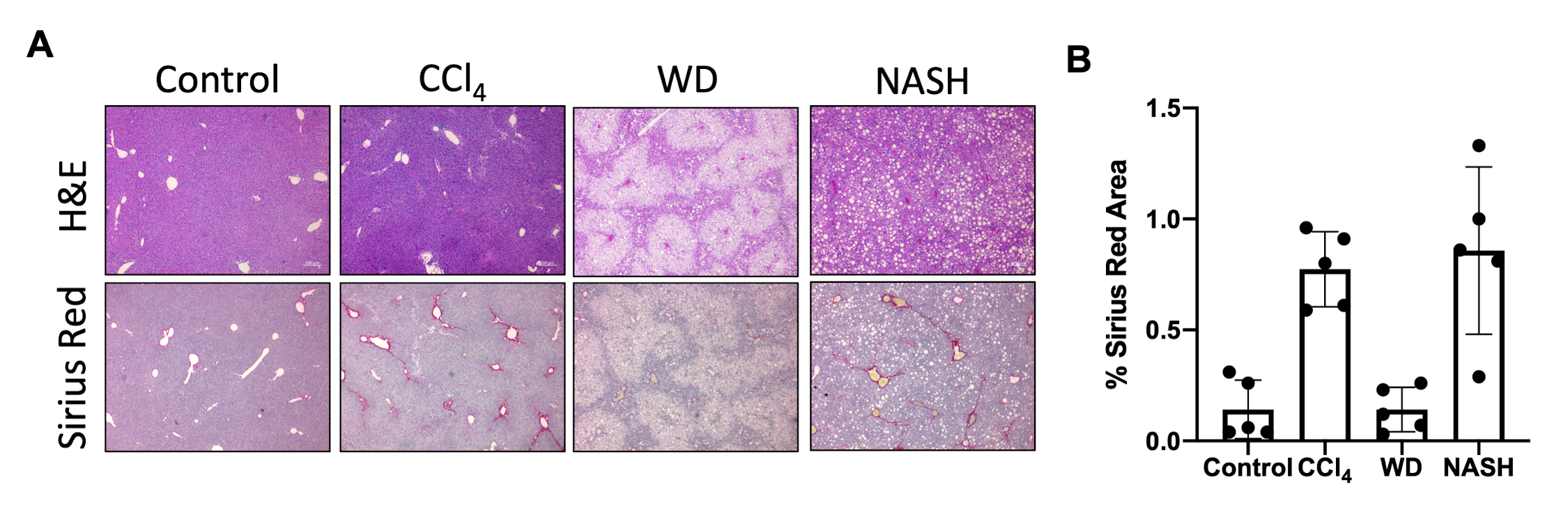

Supplement: S3 Fig — A) Liver histology assessed by H&E stain or picosirius red for fibrosis. B) Quantification of Sirius red positive area to measure extent of fibrosis. (TIFF) [file pone.0244763.s003.tiff]

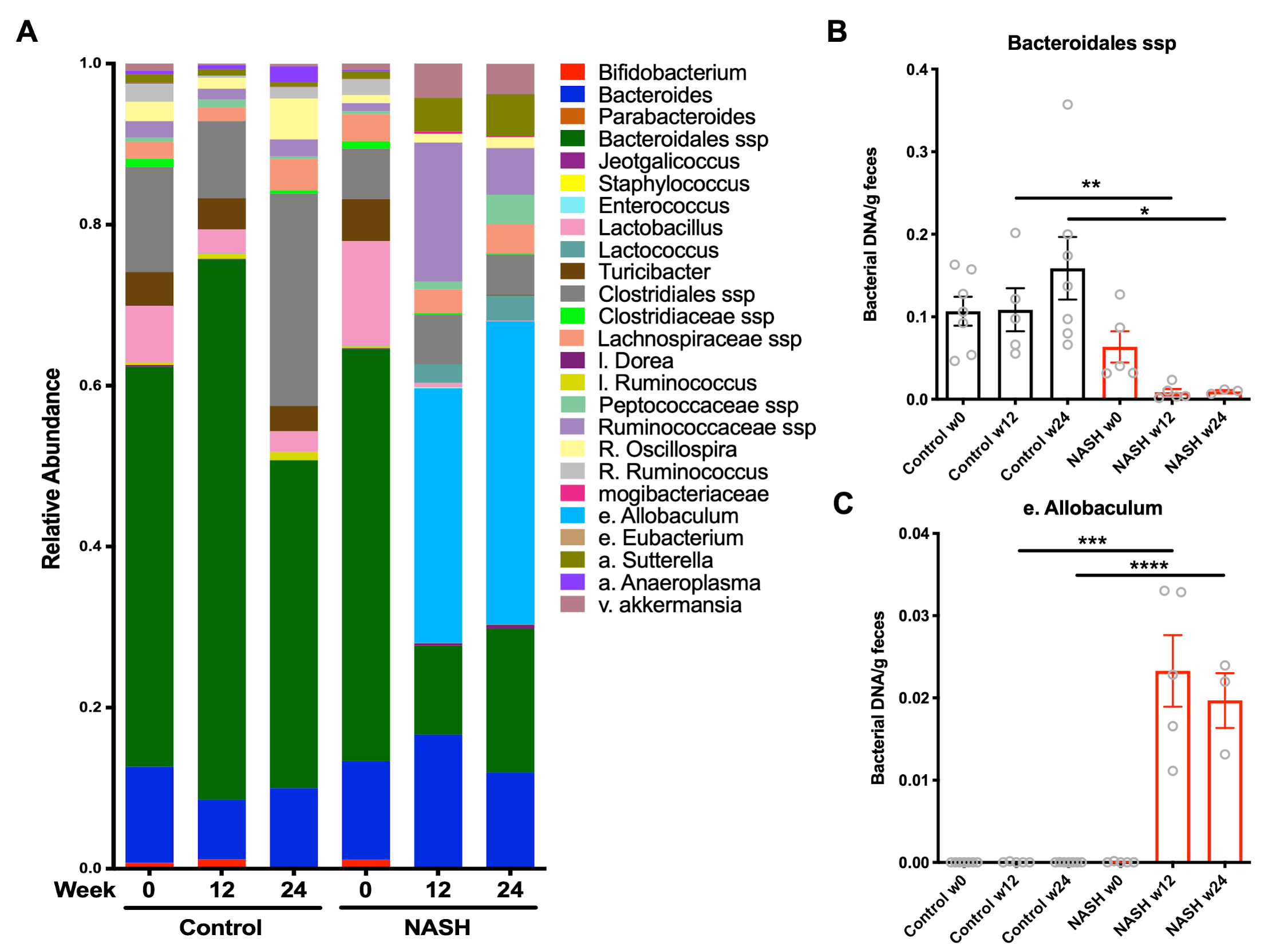

Supplement: S4 Fig — A) Genus-level relative abundance at baseline or after 12, and 24 weeks of NASH protocol. B) Bacteroidales abundance is reduced in NASH while C) Allobaculum strains from the Erysipelotrichales order increases dramatically. (TIFF) [file pone.0244763.s004.tiff]

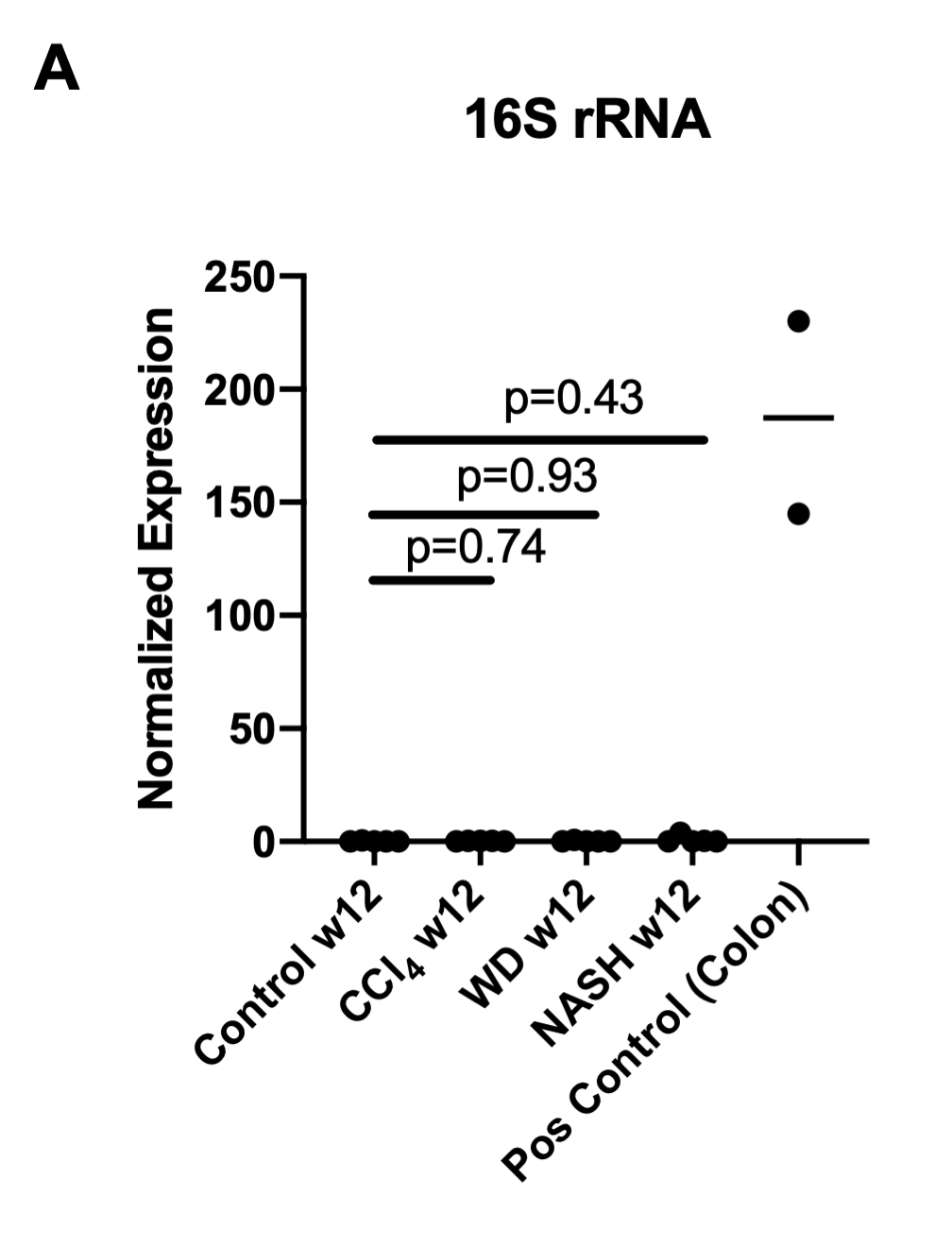

Supplement: S5 Fig — A) At week 12, RNA was extracted from whole liver tissue and screened for bacterial content by qPCR for the 16S rRNA gene in NASH and control mice. (TIFF) [file pone.0244763.s005.tiff]

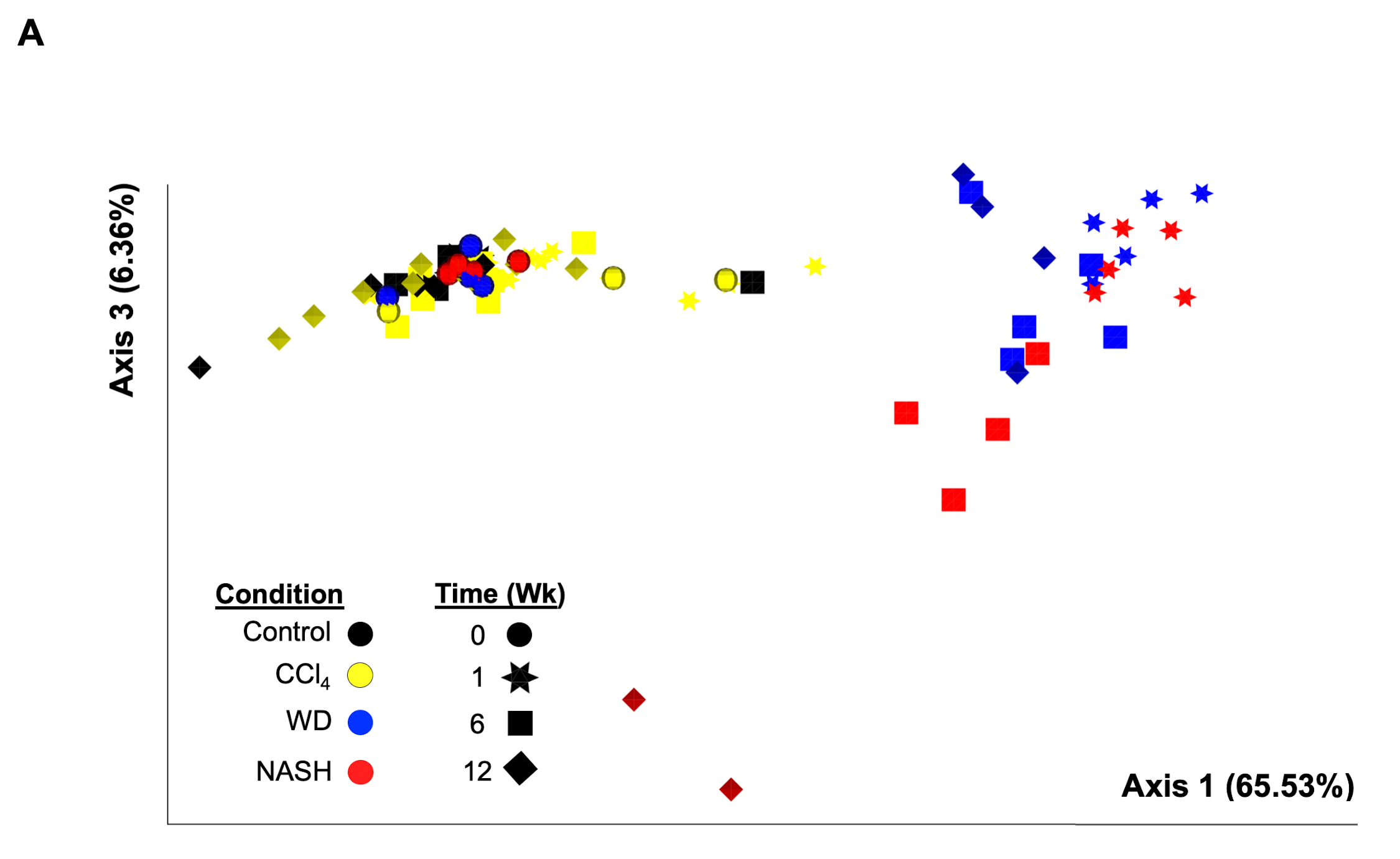

Supplement: S6 Fig — A) Principal coordinate analysis of weighted UniFrac distances demonstrates changes in bacterial composition in WD and NASH mice. (TIFF) [file pone.0244763.s006.tiff]

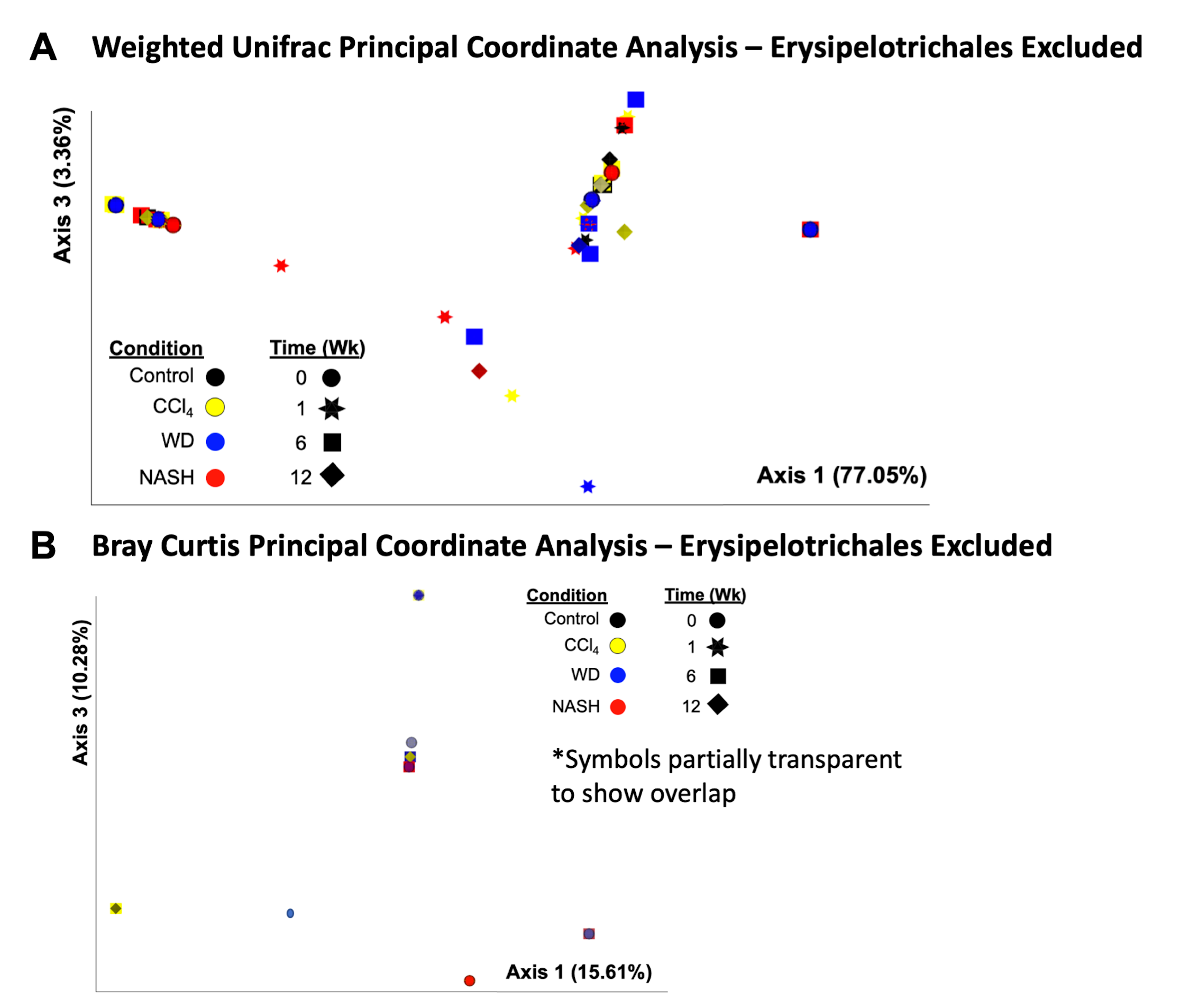

Supplement: S7 Fig — Principal coordinate analyses of beta diversity measures A) Weighted UniFrac and B) Bray-Curtis distances show microbiota from NASH and control mice no longer form distinct clusters when Erysipelotrichales removed from consideration. (TIFF) [file pone.0244763.s007.tiff]
